# Deciphering hot- and multi-exciton dynamics in core–shell QDs by 2D electronic spectroscopies†
†Electronic supplementary information (ESI) available: Description of the synthesis of QDs, experimental details, additional 2DES-PP measurements, bi-exciton analysis. See DOI: 10.1039/c8cp02574f


**DOI:** 10.1039/c8cp02574f

**Published:** 2018-07-02

**Authors:** Marcello Righetto, Luca Bolzonello, Andrea Volpato, Giordano Amoruso, Annamaria Panniello, Elisabetta Fanizza, Marinella Striccoli, Elisabetta Collini

**Affiliations:** a Department of Chemical Sciences , University of Padova , Via Marzolo 1 , I-35131 Padova , Italy . Email: elisabetta.collini@unipd.it; b CNR-IPCF SS Bari , c/o Chemistry Department , University of Bari Aldo Moro , Via Orabona 4 , I-70126 Bari , Italy; c Chemistry Department , University of Bari Aldo Moro , Via Orabona 4 , I-70126 Bari , Italy

## Abstract

Although the harnessing of multiple and hot excitons is a prerequisite for many of the groundbreaking applications of semiconductor quantum dots (QDs), the characterization of their dynamics through conventional spectroscopic techniques is cumbersome. Here, we show how a careful analysis of 2DES maps acquired in different configurations (BOXCARS and pump–probe geometry) allows the tracking and visualization of intraband Auger relaxation mechanisms, driving the hot carrier cooling, and interband bi- and tri-exciton recombination dynamics. The results obtained on archetypal core–shell CdSe/ZnS QDs suggest that, given the global analysis of the resulting datasets, 2D electronic spectroscopy techniques can successfully and efficiently dispel the intertwined dynamics of fast and ultrafast recombination processes in nanomaterials. Hence, we propose this analysis scheme to be used in future research on novel quantum confined systems.

## Abstract 2

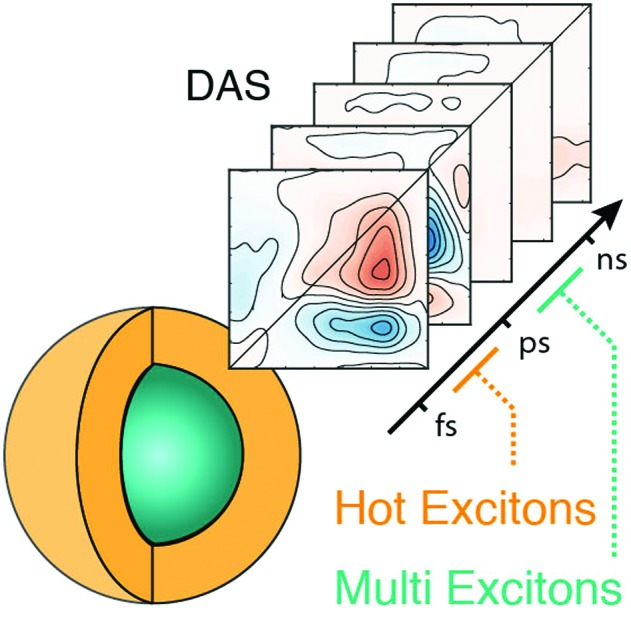
2D electronic spectroscopy maps acquired in different configurations unveil intraband hot carrier cooling and interband multi-exciton recombination dynamics.

## 

The optical properties of colloidal quantum dots (QDs) largely outpace those of conventional fluorophores.^1^,^2^ Three decades of intense research have endowed QDs with refined heterostructures, exhibiting finely controlled surface chemistry and achieving unitary quantum yields (QYs).^3^,^4^ Their size- and shape-tunable photoluminescence (PL), high oscillator strengths and enhanced photostability boosted their use in fields ranging from light emission to biological imaging.^5^–^7^ Although the technological exploitation of QDs is an already mature field, several even more promising additional applications can be envisioned. Indeed, the possibility of harnessing hot- and multi-excitons in QDs further broadens their potential, thereby leading the way to applications in photovoltaics, lasing and possibly promoting the use of QDs in quantum technologies and ultrafast multivalued logics applications.^8^–^11^

The broad absorption band of QDs allows the photoexcitation of electronically “hot” electron–hole pairs, *i.e.*, hot carriers and hot excitons (HX) containing energy in excess of the band edge energies.^12^ A fine control of the unrelenting HX cooling down to the band edge is key to a wealth of applications, such as hot carrier harvesting, optical gain, and multiple excitons generation. Applications in photovoltaics would greatly profit from slow HX cooling, which allows them to be extracted efficiently and avoid inefficiencies caused by heat dissipation. On the other hand, fast HX cooling is of fundamental importance for realizing QD-based gain media and building three-level QD-lasers.^13^,^14^ In addition, QDs support multiple excitations, *i.e.*, multi-excitons (MX), with profound implications on their practical applications. The formation of bi-exciton states (BX) influences the threshold for optical gain. In addition, their generation and recombination dynamics are deeply intertwined with HX species, through multiple excitons generation and recombination processes.^15^–^17^

Besides more conventional uses, the control of HX and MX in QDs could also represent an important prerequisite for groundbreaking applications such as ultrafast parallel multi-valued information processing, where the dynamics of these carriers may represent an additional resource to encode and process information.^18^

However, the characterization of HX and MX species is inherently complex, because it involves many coupled states and it requires sufficiently high temporal and spectral resolution to discriminate short lived and highly overlapped signals.^19^ The first experimental studies employed transient absorption (TA) and pump–push–probe spectroscopies, and more recently state-resolved TA spectroscopy provided additional insights.^10^,^20^,^21^

The introduction of selective excitations in TA spectroscopy allowed the unveiling of many details on exciton cooling and recombination in QDs. However, the increase in laser bandwidth results in a loss of excitation selectivity for pulses below 100 fs. Therefore, the QD field is still in high demand for techniques accessing both the energetics and the dynamics of elusive photophysical species, such as HXs and MXs. This gap is amplified by the incessant discovery of novel nanomaterials, such as perovskite dots and carbon dots.^22^–^25^ Two-dimensional electronic spectroscopy (2DES) has proven useful to investigate the photo-physics of different semiconductor nanomaterials.^26^,^27^ Indeed, 2DES allows the spectral resolution to be preserved while accessing high temporal resolution measurements.^19^ Although 2DES is not a standard technique to characterize QDs, it has already addressed fundamental questions such as inter-excitonic coherences, the biexcitonic fine structure, dark states, and size-dependent phonon coupling.^28^–^36^ Nevertheless, the potential of 2DES in untangling the dynamics of QDs is yet to be disclosed. For instance, the characterization of nanomaterials through 2DES will provide novel insight into fast and possibly coherent aspects of their dynamics, relevant to a wealth of applications, ranging from photovoltaics to lasing.^8^–^10^

Here, we decipher the relaxation patterns for HX and MX species in archetypal CdSe/ZnS QDs by combining results from 2DES in two different configurations: (i) BOXCARS geometry (2DES-BC) and (ii) pump and probe geometry (2DES-PP). Both configurations would allow, at least in principle, the same nonlinear signal to be investigated. However, the better time resolution achievable in the former and the higher excitation intensities reachable in the latter, make them ideal to investigate HX and MX, respectively. Thus, we chose the 2DES-BC setup to access the sub-picosecond dynamics of HX cooling, thereby operating at low excitation fluence. On the other hand, we used the 2DES-PP setup to access MX relaxations, which require high fluence and longer time windows. These measurements provide a unique visualization of HX and MX dynamics, thereby allowing the reexamination of the dynamics of these well-known QDs, in view of its application to novel and innovative nanomaterials.

We synthesized core/shell CdSe/ZnS QDs using a slightly modified version of a one-pot hot-injection method; details on the synthetic procedure and TEM images of the sample are provided in the ESI.† After the synthesis, the TOPO capped QDs were dispersed in chloroform. The size of the QDs (6.6 nm in diameter) is in good agreement with the work of Peng, considering the thickness of a few monolayers of the ZnS shell.^37^–^39^ The absorption spectrum is shown in Fig. 1a. Unlike core CdSe QDs, the absorption spectrum of CdSe/ZnS QDs is less structured due to the presence of interfacial strain between the CdSe core and the ZnS shell.^40^ Hence, we assigned the transitions underneath the absorption band by fitting them with a set of Gaussian curves (Fig. 1a), in accordance with theoretical predictions.^41^ The excitonic structure comprises four transitions in the range 15 000–19 000 cm^–1^, whose assignment is reported in the figure. According to previous studies on CdSe/ZnS QDs, the most prominent exciton peaks originate from CdSe transitions, whereas ZnS ones appear at higher energies. Therefore, the low energy transition (15 600 cm^–1^, orange curve in Fig. 1a), *i.e.*, the band-edge exciton, is ascribed to the |1S, the band-edge exciton, is ascribed to the |1S〉 or 1S or 1S_e_ – 1S_3/2_ exciton. The second transition (16 200 cm^–1^, yellow curve in Fig. 1a) is ascribed to the |2S) is ascribed to the |2S〉 or 1S or 1S_e_ – 2S_3/2_ exciton. The third transition (17 100 cm^–1^, green curve in Fig. 1a) is attributed to the |1P) is attributed to the |1P〉 or 1P or 1P_e_ – 1P_3/2_ exciton. Moreover, the estimated width of these transitions is 1000 cm^–1^ (∼120 meV) for the band-edge exciton and grows up to 1300 cm^–1^ for the |1P for the |1P〉 exciton, in agreement with the model developed by Bawendi. exciton, in agreement with the model developed by Bawendi.^42^,^43^

**Fig. 1:**
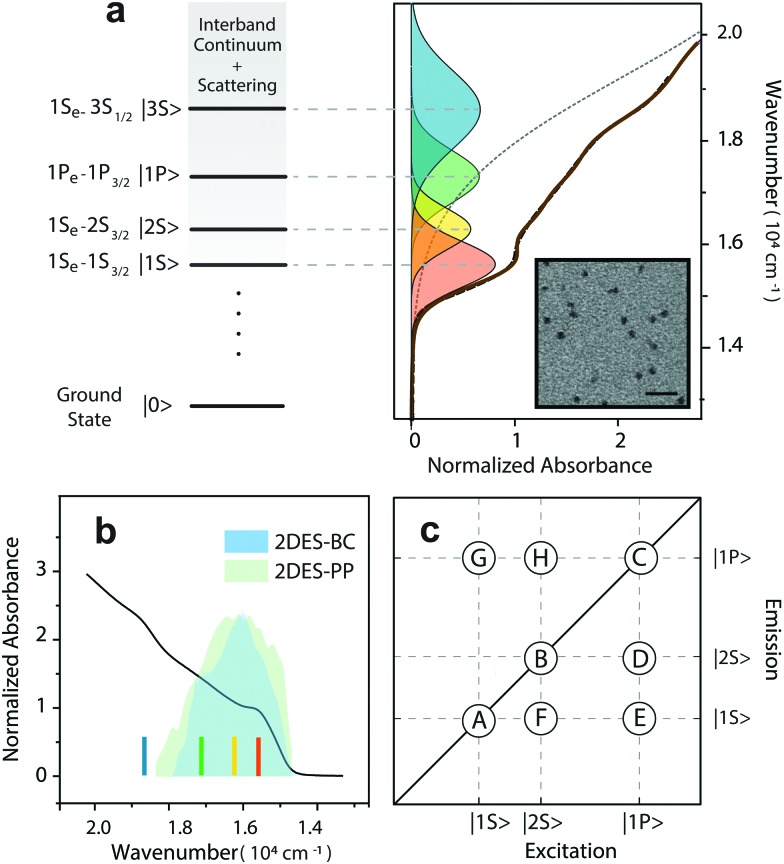
(a) Absorption spectrum of CdSe/ZnS QDs in chloroform (brown solid line). Transitions are assigned by cumulative multi-gaussian peak fitting (black dashed line). Each Gaussian peak is attributed to an exciton transition, according to ref. 43. Transmission electron microscopy (TEM) micrographs of CdSe/ZnS QDs obtained by a JEOL JEM-1011 microscope operating at 100 kV is reported in the inset (scale bar 20 nm). (b) Comparison between exciton transitions and laser bandwidth employed for the 2D experiments. Colored lines indicate the peaks of exciton transitions in the absorption spectrum. The color code is maintained across panels. (c) Coordinates of the main signals expected in the 2D maps. Diagonal signals: A (15 600, 15 600 cm^–1^), B (16 200, 16 200 cm^–1^), and C (17 100, 17 100 cm^–1^), are associated with |1S ), are associated with |1S 〉, |2S〉 and |1P〉 states, respectively. Below diagonal peaks: D (17 100, 16 200 cm, |2S), are associated with |1S 〉, |2S〉 and |1P〉 states, respectively. Below diagonal peaks: D (17 100, 16 200 cm and |1P), are associated with |1S 〉, |2S〉 and |1P〉 states, respectively. Below diagonal peaks: D (17 100, 16 200 cm states, respectively. Below diagonal peaks: D (17 100, 16 200 cm^–1^), E (17 100, 15 600 cm^–1^), and F (16 200, 15 600 cm^–1^), are associated with coupling between |2S), are associated with coupling between |2S〉 and |1P 〉, |1S〉 and |1P 〉, and |1S〉 and |2S 〉, respectively. Above diagonal signals: G (15 600, 17 100 cm and |1P ), are associated with coupling between |2S〉 and |1P 〉, |1S〉 and |1P 〉, and |1S〉 and |2S 〉, respectively. Above diagonal signals: G (15 600, 17 100 cm, |1S), are associated with coupling between |2S〉 and |1P 〉, |1S〉 and |1P 〉, and |1S〉 and |2S 〉, respectively. Above diagonal signals: G (15 600, 17 100 cm and |1P ), are associated with coupling between |2S〉 and |1P 〉, |1S〉 and |1P 〉, and |1S〉 and |2S 〉, respectively. Above diagonal signals: G (15 600, 17 100 cm, and |1S), are associated with coupling between |2S〉 and |1P 〉, |1S〉 and |1P 〉, and |1S〉 and |2S 〉, respectively. Above diagonal signals: G (15 600, 17 100 cm and |2S ), are associated with coupling between |2S〉 and |1P 〉, |1S〉 and |1P 〉, and |1S〉 and |2S 〉, respectively. Above diagonal signals: G (15 600, 17 100 cm, respectively. Above diagonal signals: G (15 600, 17 100 cm^–1^), and H (16 200, 17 100 cm^–1^) reflect the spectral coupling between |1P) reflect the spectral coupling between |1P〉, |1S〉, and |2S〉 transitions., |1S) reflect the spectral coupling between |1P〉, |1S〉, and |2S〉 transitions., and |2S) reflect the spectral coupling between |1P〉, |1S〉, and |2S〉 transitions. transitions.

The exciton transitions appear rather broad and the spectral overlap is substantial. As mentioned above, such broadening in CdSe/ZnS QDs is primarily due to inhomogeneity arising from both size dispersion and interfacial strain effects.^40^,^44^,^45^ However, 2D electronic spectroscopies allowed us to probe beneath this inhomogeneous broadening, thereby further confirming our assignments on transitions.

To investigate both HX and MX species, we adjusted the laser excitation band to cover the first three excitons (*i.e.*, |1S, |1S〉, |2S〉, and |1P〉), as shown in , |2S, |1S〉, |2S〉, and |1P〉), as shown in , and |1P, |1S〉, |2S〉, and |1P〉), as shown in ), as shown in Fig. 1b. Using 2DES-BC spectroscopy, we monitored the intraband HX dynamics, while we employed 2DES-PP spectroscopy to access the multi-exciton regime and study the MX dynamics.

We describe elsewhere the experimental setup used for 2DES-BC and report a schematic description in the ESI.† ^^46 Further details on 2D spectroscopy can be found in ref. 19 and 29. The instrumental resolution was 11 fs. During the 2DES-BC experiment, the waiting time *t*_2_ was scanned up to 2000 fs with 7.5 fs time steps. The resulting dataset is a 3D-matrix representing the evolution of 2D excitation–emission maps along the delay time *t*_2_. The temporal evolution of the purely absorptive 2D maps is shown in Fig. 2a–d. Noteworthily, the signals falling on the diagonal nicely match with the positions of transitions predicted by the multi-gaussian analysis of the absorption spectrum. Off-diagonal signals reveal the presence of spectral correlations between the different transitions.^19^

**Fig. 2:**
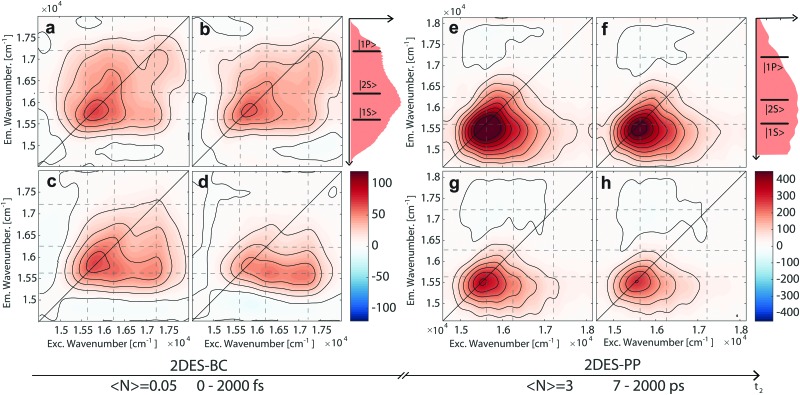
Purely absorptive 2DES-BC (a–d) and 2DES-PP (e–h) maps of CdSe/ZnS QDs in chloroform at different waiting times *t*_2_: (a) *t*_2_ = 15 fs, (b) *t*_2_ = 22.5 fs, (c) *t*_2_ = 105 fs, and (d) *t*_2_ = 1500 fs; (e) *t*_2_ = 7 ps, (f) *t*_2_ = 35 ps, (g) *t*_2_ = 280 ps, and (h) *t*_2_ = 910 ps. Dashed lines indicate the energy position of exciton transitions, as assigned in Fig. 1. On the right side of each set of panels the excitation profile and the transitions are also reported for the sake of comparison.

Qualitatively, the evolution of the 2DES-BC signal (Fig. 2a–d) sums up into two main features, describing the HX cooling process: (i) the decay of the signal in position C and the contextual growth of the signal in position E; (ii) the growth of signals in positions A, F, and E. According to the Kasha principle, the evolution of 2DES-BC maps visualizes the funneling of initially hot excitons, towards the lower-lying |1S) sums up into two main features, describing the HX cooling process: (i) the decay of the signal in position C and the contextual growth of the signal in position E; (ii) the growth of signals in positions A, F, and E. According to the Kasha principle, the evolution of 2DES-BC maps visualizes the funneling of initially hot excitons, towards the lower-lying |1S〉 exciton state. exciton state.^10^,^13^ Since the evolution of these signals involves non-independent dynamics of hot holes and hot electrons, the analysis of point time traces along *t*_2_ cannot account for the complex dynamics underneath. Hence, we analyzed the whole dataset with a global methodology to achieve an exhaustive description of the underlying dynamics, thereby avoiding problems arising from spectral crowding and signal overlap.^47^,^48^ Further details on the global analysis are provided in ref. 48 and in the ESI.† Briefly, the time evolution of the whole 2D map is fitted to a sum of complex exponentials, which allows both the incoherent dynamics associated with population decay and the coherent oscillations dephasing to be described simultaneously. Here we focus our attention only on the incoherent dynamics because the analysis of beating signals revealed no significant contributions of coherent dynamics to HX relaxation processes (see the ESI†). For each exponential component associated with a specific time constant, it is possible to map the amplitude in a 2D plot as a function of excitation and emission frequency, obtaining a decay associated spectrum (DAS).

As shown in Fig. 3a, we entirely decomposed the incoherent dynamics of CdSe/ZnS QDs into three distinct DASs. Each DAS is associated with a specific time constant (110, 340 and, ≫2000 fs, respectively) and visualizes the energetic pathways related to a process characterized by that time constant, thereby untangling correlations among evolving 2D signals. In Fig. 3b–d, we report schematically the physical interpretation of the three DASs and the expected signals on the DASs. The first DAS (*τ*_1_ = 110 fs) visualizes the hot electron cooling process though the Auger heating mechanism.^10^,^13^,^21^ As described in the level scheme of Fig. 3b, this process involves the non-radiative transfer of energy from hot electrons toward holes.^49^,^50^ The contextual decrease of the signal in position C and growth of the signals in positions D and E, proves the electron cooling from 1P_e_ to 1S_e_ and the simultaneous heating of holes from 1P_3/2_ to higher-lying hole states (*e.g.*, 2S_3/2_). Indeed, due to the Pauli principle and state filling effect, the increased 1S_e_ state population results in enhanced bleaching of both |1S state population results in enhanced bleaching of both |1S〉 and |2S〉 exciton transitions. and |2S state population results in enhanced bleaching of both |1S〉 and |2S〉 exciton transitions. exciton transitions.^13^,^41^ The coupled growth and decay of the signal in positions C, D and E, respectively, are the most evident features sketched in Fig. 3b. However, a weaker low energy signal is observed, below positions A, F, and E. This signal is related to the “bi-exciton shift” effect: indeed, the cooling of hot electrons in hot holes is expected to be related to this photo-induced absorption signal.

**Fig. 3:**
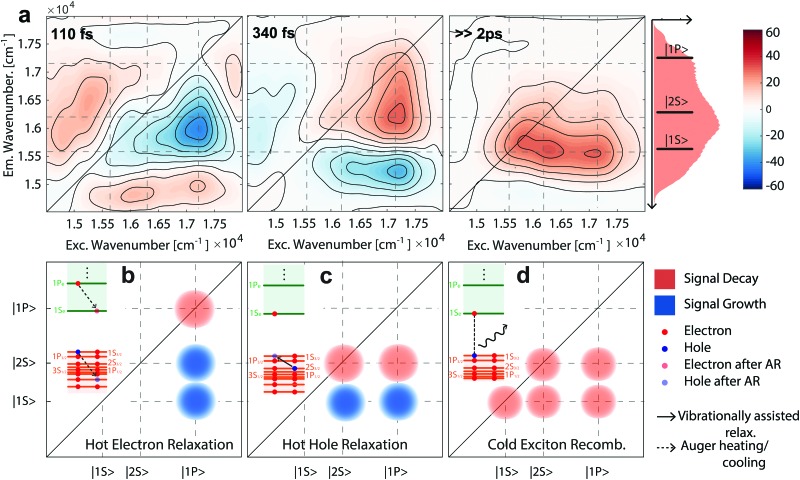
(a) Global analysis fitting results for the 2DES-BC spectrum of CdSe/ZnS QDs. The dynamics is decomposed into three DASs associated with time constants of 110 fs, 340 fs and ≫2 ps, respectively. The amplitude of the third DAS is divided by a factor of two to ease the comparison with the other panels. Description of the hot electron (b), hot hole (c), and cold exciton (d) relaxation and positions at which signals are expected in each DAS.

Noteworthily, the subsequent hot hole cooling is observed in the second DAS (*τ*_2_ = 340 fs), where the signal decays in positions D and B and grows contextually in positions E and F. This transfer represents the cooling of hot holes from the 2S_3/2_ to the lower lying 1S_3/2_ state. Lastly, the third DAS (*τ*_3_ ≫ 2000 fs) accounts for the slower relaxation of band-edge excitons. According to the shape of the signal, both the band-edge exciton and cooled HX contribute to this relaxation, taking place on longer timescales. Thus, the spectral decoupling provided by 2DES allows visualization of the HX relaxation process directly.^21^

The assignment of 110 and 340 fs time constants to hot electron and hot hole relaxations, respectively, is consistent with previous reports.^13^,^51^ Quantitatively, an analogous hot electron cooling time constant (100 fs) was recently reported in thiol capped CdSe QDs.^31^ In core QDs, ligands appear as central in the relaxation of hot electrons, thereby suggesting the presence of a surface related relaxation channel.^52^ However, in our core–shell QDs, the ZnS shell decouples the electronic states of CdSe from the surface and therefore ensures that we are observing the Auger-mediated relaxation dynamics. This decoupling from the surface state is observed in the hot hole dynamics, as well. Although previous studies reported size independent ∼250 fs hole cooling in CdSe QDs, we observe a slower dynamic. Again, the surface passivation by ZnS, inhibits the surface related hole relaxation pathways (*e.g.*, coupling with phonons at surfaces and ligands) and slows down the recombination.^53^ Even though TA cannot access directly spectral couplings, Kambhampati *et al.* demonstrated the possibility of decoupling these processes by comparison of the dynamics of state-resolved TA transients.^13^,^51^,^53^ Specifically, the hot hole dynamics was uncovered by comparing the dynamics of a photoinduced absorption signal below the band-edge (referred to in the literature as A1), under |1S Specifically, the hot hole dynamics was uncovered by comparing the dynamics of a photoinduced absorption signal below the band-edge (referred to in the literature as A1), under |1S〉 and |2S〉 state resolved excitation, respectively. and |2S Specifically, the hot hole dynamics was uncovered by comparing the dynamics of a photoinduced absorption signal below the band-edge (referred to in the literature as A1), under |1S〉 and |2S〉 state resolved excitation, respectively. state resolved excitation, respectively.^13^ Notably, we observe dynamics of signals below positions A, F and E (emission freq. 15 300 cm^–1^) in correspondence with the A1 signal previously studied by state resolved TA spectroscopy. The observed growth with *τ*_1_ = 110 fs and decay with *τ*_2_ = 340 fs time constants at position (16 200, 15 300) cm^–1^ is in good agreement with previous studies and further confirms our assignments.^13^,^51^,^53^

Differently from HX, the MX species in CdSe QDs usually undergo relaxation within tens or hundreds of picoseconds.^17^,^54^ We characterized these dynamics by power-dependent 2DES-PP measurements, scanning *t*_2_ throughout the 0–2000 ps time interval with 7 ps steps. 2DES-PP measurements were performed using a 2D setup in pump–probe geometry following a previously reported procedure (further details in the ESI†).^55^,^56^ To access the multiexcitonic regime, we excited QDs at different fluences *j*, corresponding to different average exciton occupancies within the ensemble (*i.e.*, , 〈*N*〉 = = *j* = 0.3, 1.2, 3, 4 electron–hole pairs). The initial distribution of excitons per dot after a short laser pulse follows Poissonian statistics (Fig. SI_11, ESI†).^41^

In Fig. 2e–h, we report the evolution of recorded 2DES-PP maps at increasing population times, under , we report the evolution of recorded 2DES-PP maps at increasing population times, under 〈*N*〉 = 3 e–h fluence (maps recorded at different fluence values are reported in Fig. SI_4, ESI = 3 e–h fluence (maps recorded at different fluence values are reported in Fig. SI_4, ESI†). Qualitatively, the shape of the positive signal in positions A, F, and E is elongated horizontally and resembles that of the third DAS in Fig. 3a, thereby representing the completion of the cooling dynamics. In addition, we observe a broad above-diagonal negative feature in positions G and H, persisting throughout the entire measurement. Fig. 4d reports the transients of the position A: with increasing excitation fluence, we observe faster recombination dynamics, ascribed to the generation of multiple exciton states. According to Klimov, we interpret our signals as generated by a statistical mixture of dots bearing a different number of excitons, following a Poisson statistics.^41^ As shown in Fig. SI_11 (ESI†), even at the lowest fluence used (*i.e.*, 0.3 e–h), we are generating a non-negligible fraction of BX. With increasing excitation fluence, the probability of generating BX and tri-excitons (TX) increases further.

**Fig. 4:**
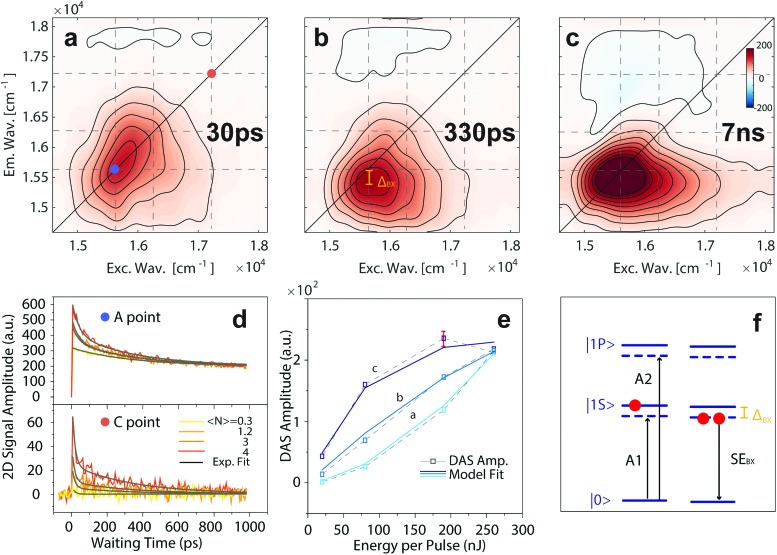
(a–c) Results of the global fitting analysis for the 2DES-PP measures on CdSe/ZnS QDs; the dynamics is decomposed into three DASs. The associated time constants are reported in each panel (30, 330 ps and 7 ns, respectively). (d) Power dependence of the signal recorded at coordinates *A*, and *C*, ascribed to the |1S, ascribed to the |1S〉 and |1P〉 population, respectively. (e) Model fitting of the power dependent amplitudes for the three DASs reported in the upper panels. The plotted error bar was estimated through the analysis of two different datasets. (f) Energy levels of the typical signal arising from the single and bi-exciton population in the QDs. Adapted from and |1P, ascribed to the |1S〉 and |1P〉 population, respectively. (e) Model fitting of the power dependent amplitudes for the three DASs reported in the upper panels. The plotted error bar was estimated through the analysis of two different datasets. (f) Energy levels of the typical signal arising from the single and bi-exciton population in the QDs. Adapted from population, respectively. (e) Model fitting of the power dependent amplitudes for the three DASs reported in the upper panels. The plotted error bar was estimated through the analysis of two different datasets. (f) Energy levels of the typical signal arising from the single and bi-exciton population in the QDs. Adapted from ref. 13 and 15.

Aiming to untangle the wealth of information laying underneath the observed signal, we extended the global fitting approach used for 2DES-BC by including the power dependence. Hence, we performed a global fitting considering jointly the four datasets recorded under different fluences (further details in the ESI†). The rationale underlying this approach considers that when increasing the fluence, we are acting only on the ratio of multiexcitonic species generated and not on their dynamics. Thus, the observed dynamics is originated by a definite number of processes (*i.e.*, exciton, BX, and TX recombinations), whose relative amplitude should follow a Poissonian behavior.^57^

Fig. 4a–c report the three DASs obtained by global fitting of the 2DES-PP map recorded under report the three DASs obtained by global fitting of the 2DES-PP map recorded under 〈*N*〉 = 3 e–h fluence. Noteworthily, each of the three processes contributes to the recombination in different regions of the spectrum. Considering its time constant and the amplitude distribution, we assign the third DAS ( = 3 e–h fluence. Noteworthily, each of the three processes contributes to the recombination in different regions of the spectrum. Considering its time constant and the amplitude distribution, we assign the third DAS (Fig. 4c) to singly excited QD recombination, largely outranging the investigated time interval. This DAS presents signals at diagonal coordinates A and weaker signals in position F and E, thereby indicating the recombination of cooled excitons with a slow time constant (*τ*_6_ = 7 ns), consistently with the radiative time constant determined through time-resolved photoluminescence (Fig. SI_2, ESI†). Moreover, we observe a negative and broad above-diagonal signal across positions G and H, which nicely displays the so-called “bi-exciton effect”.^15^ Namely, the population of the |1S Namely, the population of the |1S〉 state generates an energy level shift state generates an energy level shift *via* Coulombic interactions, thereby giving rise to photoinduced absorption (PIA) signals, labelled as A1 and A2 in Fig. 4f, in agreement with ref. 10, 15 and 50. The out-of-diagonal nature of this signal confirms that the A1 and A2 signals stem from level shifting effects, as sketched in Fig. 4f.

Although the shape of the signals observed in the second DAS (Fig. 4b) resembles that of the third DAS, the signal is redshifted and located out of the diagonal. Hence, considering its faster dynamics (*τ*_5_ = 330 ps), we safely attribute it to BX recombination. Indeed, as shown in Fig. 4f, the stimulated emission signal is redshifted with respect to the exciton absorption by the stabilizing interaction energy between two excitons in QDs, *i.e.*, the bi-exciton binding energy (*Δ*_BX_). Analogous measurements were performed with a redshifted excitation laser band to quantitatively extract the *Δ*_BX_ value *Δ*_BX_ = 190 ± 10 cm^–1^ (see the ESI†).^15^ Generally, the BX species were characterized using TA by pumping at low fluences and monitoring the biexciton induced shift of |1S Generally, the BX species were characterized using TA by pumping at low fluences and monitoring the biexciton induced shift of |1S〉 absorption (A1 signal according to Klimov). absorption (A1 signal according to Klimov).^41^,^58^,^59^ However, due to the high fluence employed in our 2DES-PP experiments we access directly the BX stimulated emission signal. BX is known to undergo relaxation *via* an Auger process, and hence trapped charges and interface potentials and the dimensions play a pivotal role in the dynamic behavior. A recent report on BX recombination by Kelley in CdSe/ZnSe QDs is in fair agreement with our results, suggesting the absence of trap-mediated Auger BX recombination in our samples.^60^

The direct observation of higher MX recombination (*i.e.*, TX and so forth) by TA was emphatically debated within the scientific community. Due to the concurrence of fast surface trapping processes and ensuing possible photocharging effects,^61^ the univocal determination of MX dynamics is a difficult task.^17^ Considering the double degeneracy of the |1S Considering the double degeneracy of the |1S〉 state, our laser bandwidth ( state, our laser bandwidth (Fig. 1c) gives us access to the generation of TX, through the excitation of |1P) gives us access to the generation of TX, through the excitation of |1P〉 excitons. However, TX is expected to have little impact on the |1S〉 transition, but rather to influence higher energy transitions. excitons. However, TX is expected to have little impact on the |1S) gives us access to the generation of TX, through the excitation of |1P〉 excitons. However, TX is expected to have little impact on the |1S〉 transition, but rather to influence higher energy transitions. transition, but rather to influence higher energy transitions.^15^,^62^ The rather broad signal observed in the first DAS is peaked at (16 300, 16 300) cm^–1^ and recombines with fast dynamics, *τ*_4_ = 30 ps.^54^ Even though similar recombination times were attributed to hole trapping in CdSe QDs, a detailed analysis of the 2DES-PP map allows this process to be ascribed univocally to the recombination of TX species. Fig. 4d reports the fluence dependent trace of the position C, associated with the population of |1P reports the fluence dependent trace of the position C, associated with the population of |1P〉 states. Notably, this signal grows with fluence, following the Poisson distribution of excitation. Hence, a non-zero |1P〉 population on the picosecond timescale is the direct signature of a tri-exciton. As reported in the ESI, states. Notably, this signal grows with fluence, following the Poisson distribution of excitation. Hence, a non-zero |1P reports the fluence dependent trace of the position C, associated with the population of |1P〉 states. Notably, this signal grows with fluence, following the Poisson distribution of excitation. Hence, a non-zero |1P〉 population on the picosecond timescale is the direct signature of a tri-exciton. As reported in the ESI, population on the picosecond timescale is the direct signature of a tri-exciton. As reported in the ESI,† in conventional TA measurement this signal is hidden underneath the PIA band generated by level shifting.^41^ Lastly, the observed dynamic does not follow the scaling laws described by Klimov for CdSe QDs.^63^ We attribute this discrepancy to the presence of a sharp CdSe/ZnS interface, possibly enhancing Auger interactions in the TX multiparticle complex.^45^,^60^,^64^

To further confirm our assignments, we performed a model-based analysis of power dependent amplitudes for the three different DAS reported in Fig. 4a–c. Namely, we extended the global fit by including time as well as the power coordinate (Fig. 4d). The power dependent behavior reveals a saturation trend for the DAS related to exciton and BX species, caused by the state filling of the twofold degenerate |1S). The power dependent behavior reveals a saturation trend for the DAS related to exciton and BX species, caused by the state filling of the twofold degenerate |1S〉 exciton state. Noteworthily, according to the universal curve by Klimov, the slower saturation of biexciton species ( exciton state. Noteworthily, according to the universal curve by Klimov, the slower saturation of biexciton species (*E*_sat,X_ = 47 nJ; *E*_sat,BX_ = 158 nJ) can be ascribed to a reduction of the absorption cross section for BX, as previously reported by Pullerits.^41^,^57^ Further details on the power-dependent global fitting can be found in the ESI.† On the other hand, no saturation is observed for the DAS related to TX, due to the involvement of a highly degenerate |1P On the other hand, no saturation is observed for the DAS related to TX, due to the involvement of a highly degenerate |1P〉 state. state.

In conclusion, 2DES proves to be a key technique for a comprehensive characterization of HX and MX in QDs. Hitherto, the characterization of these species required multiple or complicated measurements based on the TA technique. In this Letter, we proved how the combination of high temporal and energetic resolution provided by 2DES-BC dispels with ease the intricate HX cooling mechanisms. Using global analysis, we were able to disentangle and observe the concerted hot electron and hot hole cooling directly, driving HX cooling *via* the Auger mechanism and vibrational coupling, respectively. Analogously, through the application of global analysis methodologies, we revealed the univocal signatures of BX and TX in 2DES-PP measurements under higher intensity excitations. The consideration of the DAS, as well as their power dependent behavior, allowed each process to be assigned univocally. Ultimately, we demonstrated that 2DES measurements wholly contain the same information gathered along three decades of research on QDs. We believe that the proposed characterization will be particularly advantageous in the study of how ligands and shell effects (*i.e.*, concerning traps, interfacial strain, shell potential and confinement) subtly influence the HX and MX dynamics, to improve the rational design of QD systems. Hence, dealing with the complexity of QD exciton and multi-exciton dynamics, we propose the 2DES techniques as a helpful tool in the research on novel nanomaterials.

## Conflicts of interest

There are no conflicts of interest to declare.

## Supplementary Material

Supplementary information
