# Supplementary material for: Deciphering hot- and multi-exciton dynamics in core–shell QDs by 2D electronic spectroscopies
Source: Phys Chem Chem Phys. 2018 Jul 2;20(27):18176–83. doi: 10.1039/c8cp02574f (PMC6044327; doi:10.1039/c8cp02574f)
Supplement: Supplementary file 1 [file CP-020-C8CP02574F-s001.pdf]

Electronic Supplementary Material (ESI) for Physical Chemistry Chemical Physics.
This journal is © the Owner Societies 2018

Deciphering Hot- and Multi-Exciton Dynamics in Core-Shell QDs by 2D Electronic Spectroscopies

*Marcello Righetto†, Luca Bolzonello†, Andrea Volpato†, Giordano Amoruso†, Annamaria Panniello‡, Elisabetta Fanizza§‡, Marinella Striccoli‡, Elisabetta Collini†**

†Department of Chemical Science, University of Padova, Via Marzolo 1, 35131 Padova, Italy

‡CNR-IPCF-Bari Division, c/o Chemistry Department, University of Bari Aldo Moro, Via Orabona 4, 70126 Bari, Italy

§Chemistry Department, University of Bari Aldo Moro, Via Orabona 4, I-70126 Bari, Italy

Email: elisabetta.collini@unipd.it

SUPPORTING INFORMATION

Contents:

- **Synthesis and basic characterization of CdSe/ZnS QDs** S2
- **2DES BOXCARS experimental setup** S4
- **2DES Pump & Probe experimental setup** S5
- **Global fitting of 2DES-PP data** S6
- **Coherent Dynamics in 2DES-BC data** S10
- **Additional 2DES-PP measurements** S11
- **Biexciton Binding Energy** S13

Synthesis and basic characterization of CdSe/ZnS QDs

CdSe/ZnS QDs were synthesized under nitrogen flux adapting a previously *one pot* reported procedure.[1,2] All solvents were of the highest purity available and used as received. CdO powder, trioctylphosphine oxide (TOPO) (technical grade 90%), trioctylphosphine (TOP), hexadecylamine (HDA, 90%), tetrabutylphosphonic acid (TBPA, 97%), hexamethyldisilathiane (HMST), and diethylzinc (1 M solution in hexane) were purchased from Sigma-Aldrich. Typically, a mixture of TOPO (9.7 g), HDA (9.7 g), TBPA (0.276 g) and CdO (0.127 g) are dried and degassed in a 50 mL three-neck flask under vacuum by vigorous stirring at 120°C for about 20 min and afterwards heated in N_2 atmosphere up to 300°C. As the temperature reaches 290°C, TBP (2mL) is added to the reaction mixture. The nucleation step is gained by the fast injection of the Se precursor solution, obtained by dissolving elemental selenium (0.394 g) in TBP (4.4 g), into the hot mixture at 300°C. The desired QD size is achieved by further growing at 270°C for variable reaction time. After an annealing step at 110°C for 60 min, the inorganic shell of ZnS is directly grown on the CdSe nanocrystal core. The stock solution of ZnS precursors is prepared in a glove box by mixing $Zn(C_2H_5)_2$ (1.294 g), HMDS (0.380 g) and TBP (9.460 g). The proper volume of such solution is added by a drop-wise injection at 155°C in the original reaction flask. The shell growth is controlled in real-time by monitoring the UV-vis absorption and emission spectra of the solution. The CdSe/ZnS QD dispersion is cooled to 110°C and further stirred under N_2 flux at this temperature for 60 min, to stop the shell growth. Finally, the temperature is decreased at 60°C and the QDs are washed by repeated precipitation with methanol and finally re-dispersed in a small volume of chloroform.

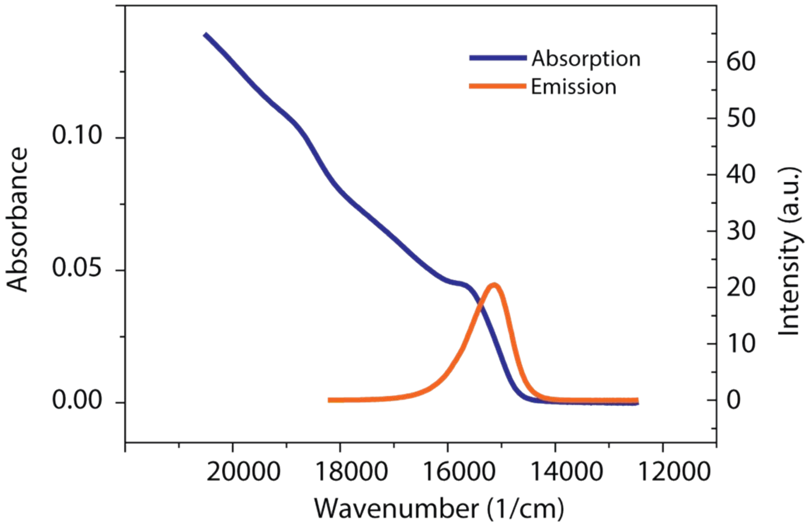

Figure SI_1- Absorption (blue line) and photoluminescence (orange line) spectra of CdSe/ZnS QDs in chloroform solution.

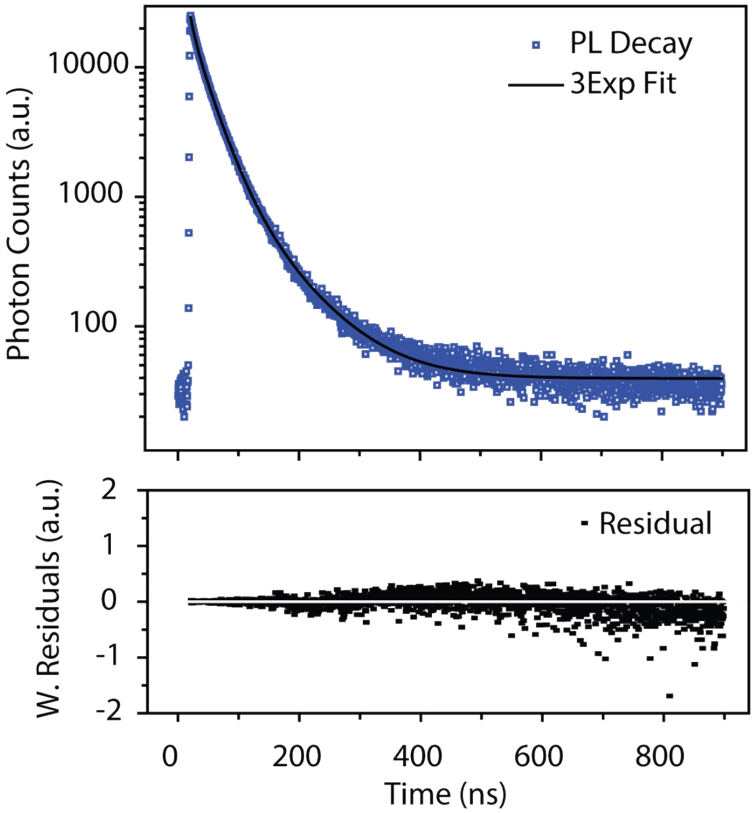

Figure SI_2- Time Resolved Photoluminescence spectrum of CdSe/ZnS QDs in solution (blue dots) and tri-exponential fitting curve (black line). Excitation 365 nm, Emission 640 nm. The lower box reports weighted residuals from tri-exponential fitting.

In Figure SI_1, we report the absorption and steady-state photoluminescence (PL) spectra of the studied QDs in chloroform solution. As well, we performed time resolved photoluminescence measurements on the nanosecond timescale by time correlated single photon counting methods. The PL decay curve recorded under 365 nm blue excitation [1.3 ns resolution, 1 MHz repetition rate] is reported in Figure SI_2. The PL dynamics is well described by a tri-exponential function with time constants $\tau_A = 7\ ns$, $\tau_B = 27\ ns$, and $\tau_C = 75\ ns$ and normalized amplitudes 72%, 25%, and 3%, respectively. The weighted residuals analysis confirms the absence of residual small amplitude dynamics.

2DES BOXCARS experimental setup

2DES-BC measurements have been performed with the setup described in Ref [3]. The pulses energy at the sample position was reduced down to 5 nJ per pulse, by a broadband half-waveplate/polarizer system while the beam waist was 300 μm. This lead to a <N> = 0.05. t_2 was scanned from 0 to 2000 fs in steps of 7.5 fs and for each value of t_2, the coherence time t_1 was scanned from 0 to 80 fs in steps of 1 fs. To characterize the temporal properties of the exciting pulses, we performed FROG measurements (Figure SI_3) in the same experimental conditions of 2DES measurements, only replacing the QDs solution with the solvent in the same 1 mm path-length cuvette.

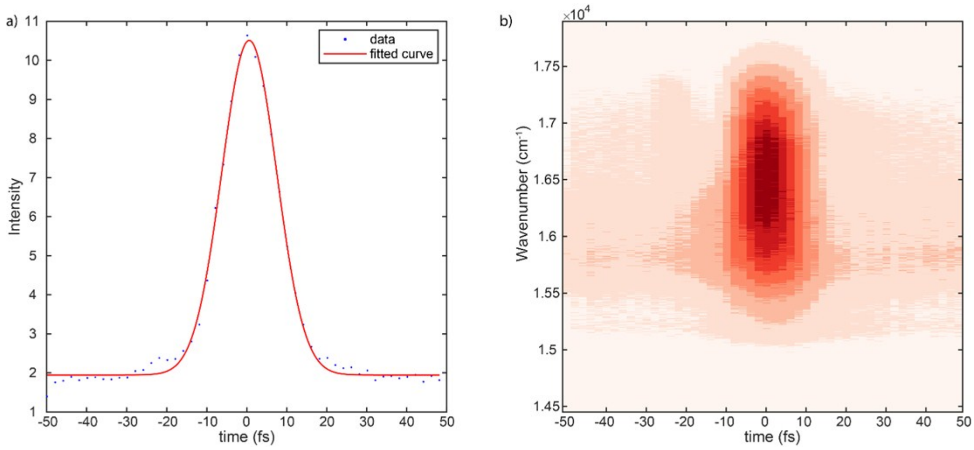

Figure SI_3- (**a**) FROG signal integrated along the frequency axis. The experimental curve (blue dots) is well fitted by a gaussian function with FWHM of 16 fs (red line), leading to a pulse duration of 11.5 fs. (**b**) FROG signal resolved in frequency highlights the absence of significant phase distortions in the pulse.

We retrieved a pulse duration of 11.5 fs, reasonably assuming gaussian pulses (see Figure SI_3).[4] To phase the signal, we compared BC spectra with 2DES-PP spectra recorded at the same pump energy.

The storage and handling of samples was conducted under N_2 atmosphere. During measurements, QDs were kept under N_2 atmosphere. To ensure the absence of any photodegradation process, we performed comparison between the linear absorption spectrum of QDs solutions before and after the measurements. At least 3 different sets of measurements in different days were performed and then

averaged to ensure the reliability and reproducibility of the reported measurements. All measurements were performed at ambient temperature (295 K).

2DES Pump & Probe experimental setup

2DES-PP experiment was performed using the optical layout already described in Refs[5,6] and reported in Figure SI_4.

Similarly to 2DES-BC, also in 2DES-PP the laser pulse was generated by a Ti:Sapphire laser-NOPA coupled system. After the NOPA, the beam was collimated and divided by a beam splitter. The 10% of the beam was used as the probe pulse, whose chirp was minimized through a prism compressor; it was then focused by spherical lenses, delayed with a retroreflector mounted on an Aerotech ATS 100 linear stage and finally sent to the sample. The remaining 90% was used to generate the two collinear pump pulses through pulse shaping techniques.[6] An acousto-optic programmable dispersive filter (AOPDF, Fastlite® Dazzler) was indeed used to generate two replicas of the incoming beam and impose increasing values of delay time between them. Again, a prism compressor was used to control the temporal chirp. Pump pulses were then collimated by spherical mirrors and finally focused on the sample in the same position of the probe. 2DES-PP maps were retrieved scanning t_2 from 0 to 2 ns in steps of 7 ps; for each value of t_2 the coherence time t_1 was varied from 0 to 80 fs in steps of 1 fs. Well-established phase cycling techniques [Refs 5,6] have been used to retrieve rephasing and non-rephasing signals. However, pure absorptive signal (rephasing + non-rephasing) is used to study the dynamics of QDs.

The beam waist of pump beam was 300 μm. The energies of pump pulses used for power dependence measurement were 20, 80, 190 and 270 nJ associated with an average exciton occupancy following Poisson distribution ($\langle N \rangle$) of 0.3, 1.2, 3 and 4, respectively.

The storage and handling of samples was conducted under N_2 atmosphere. During measurements, QDs were kept under N_2 atmosphere. To ensure the absence of any photodegradation process, we

performed comparison between the linear absorption spectrum of QDs solutions before and after the measurements.

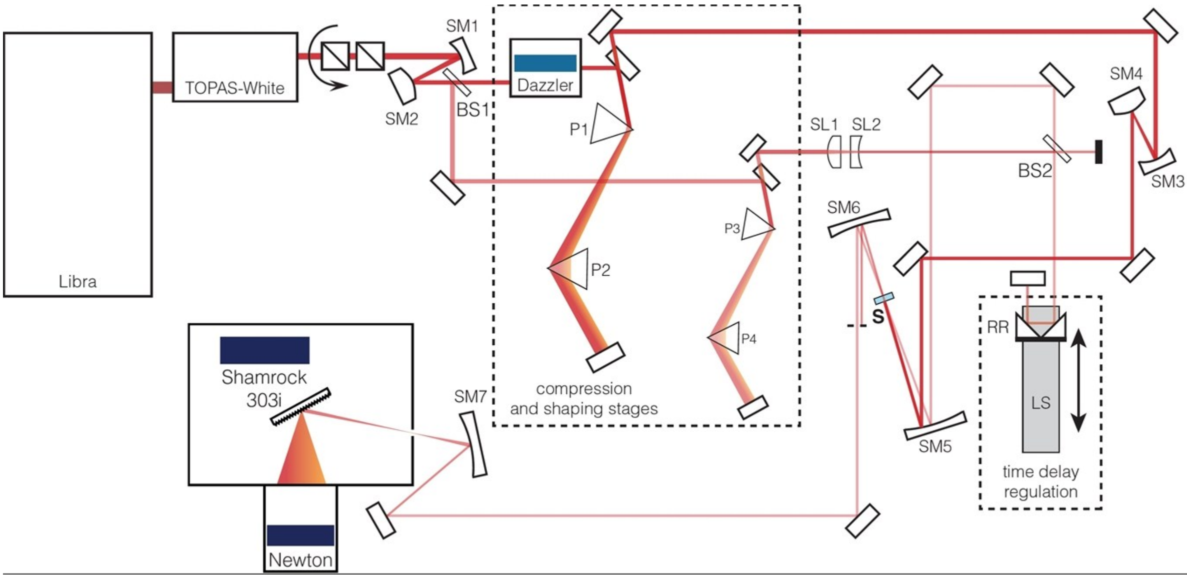

Figure SI_4 – The optical layout of the 2DES-PP experiment.

Global Analysis

Both 2DES-BC and 2DES-PP signals were analyzed with a global complex multi-exponential fitting procedure, following a recently proposed methodology.[7] Briefly, the decay of the total complex signal at each point of the 2D map is fitted with a global function written as a sum of complex exponentials:

$$f(t_2) = \sum_{n=1}^{N} a_n e^{i\phi_n} e^{-t_2/\tau_n} e^{i\omega_n t_2} \qquad \text{S1}$$

where ω_n are oscillation frequencies, τ_n are decay constants, ϕ_n are phases and a_n are signal amplitudes.

The components with $\omega_n = 0$ describe population decay contributions, whereas components with $\omega_n \neq 0$ represent oscillating components, associated with the coherent dynamics along t_2. The

corresponding amplitude a_n plotted in a 2D map as a function of ν_1 and ν_3 builds the so-called DAS (decay associated spectra) and CAS (coherence associated spectra), respectively. These maps allow the direct visualization of both sign and the amplitude distribution of a particular decay component along the 2D spectra. Given the n-*th* DAS associated with the time constant τ_n, a positive amplitude (red areas) is found at positions in the 2D maps where the signal is decaying with τ_n, whereas a negative amplitude (blue areas) is found where the signal is rising with τ_n.

A further stage of global analysis studied the dependence of 2DES-PP DAS as a function of pulse energy.

For each time constant identified in the first step of the global analysis, four different DAS can be drawn, each one corresponding to a different value of pulse energy (see columns in Figure SI_5). It is thus possible to assemble 'bundles' of DAS, consisting of 3D matrices showing the evolution of the 2D frequency-frequency DAS as a function of the pulse energy e.

These DAS bundles (one for each time constant) are then globally fit with a procedure entirely analogous to the one already described in Eq. S1, except that the variable, in this case, is the pulse energy e rather than the delay time t_2. The fitting function follows an effective model reproducing the rising and the saturation of the signals:

$$f'(e) = a_{sat} f_{sat}(e) + a_{ris} f_{ris}(e) \qquad \text{S2}$$

where the saturation term is expressed as $f_{sat}(e) = 1 - \left(1 + \frac{ke}{2}\right)e^{-ke}$ in agreement with Ref. 8, and the rising term is expressed as $f_{ris}(e) = e^b$.

a_{sat} and a_{ris} are the amplitudes of the two components, k is a coefficient tuning the saturation process such that $<N> = ke$, and b is the exponent modulating the rising component.

The results of the fitting are reported in Table S1 and correspond to the trend shown in Figure 4e of the main text. DAS 2 shows a residual TX signature arising from the non-complete dynamics separation of the multi-exponential model. However, a perfect separation of these contributions was achieved by global analysis of the power coordinate.

Table S1. Results of the global fitting performed applying Eq.S2 to the DAS bundles. Errors are estimated from the standard error of the minimization problem.

DAS #	time constant	k (nJ^{-1})	b
1	30 ps	\	1.63±0.02
2	330 ps	0.006±0.005	1.74±0.04
3	7 ns	0.0213±0.0003	\

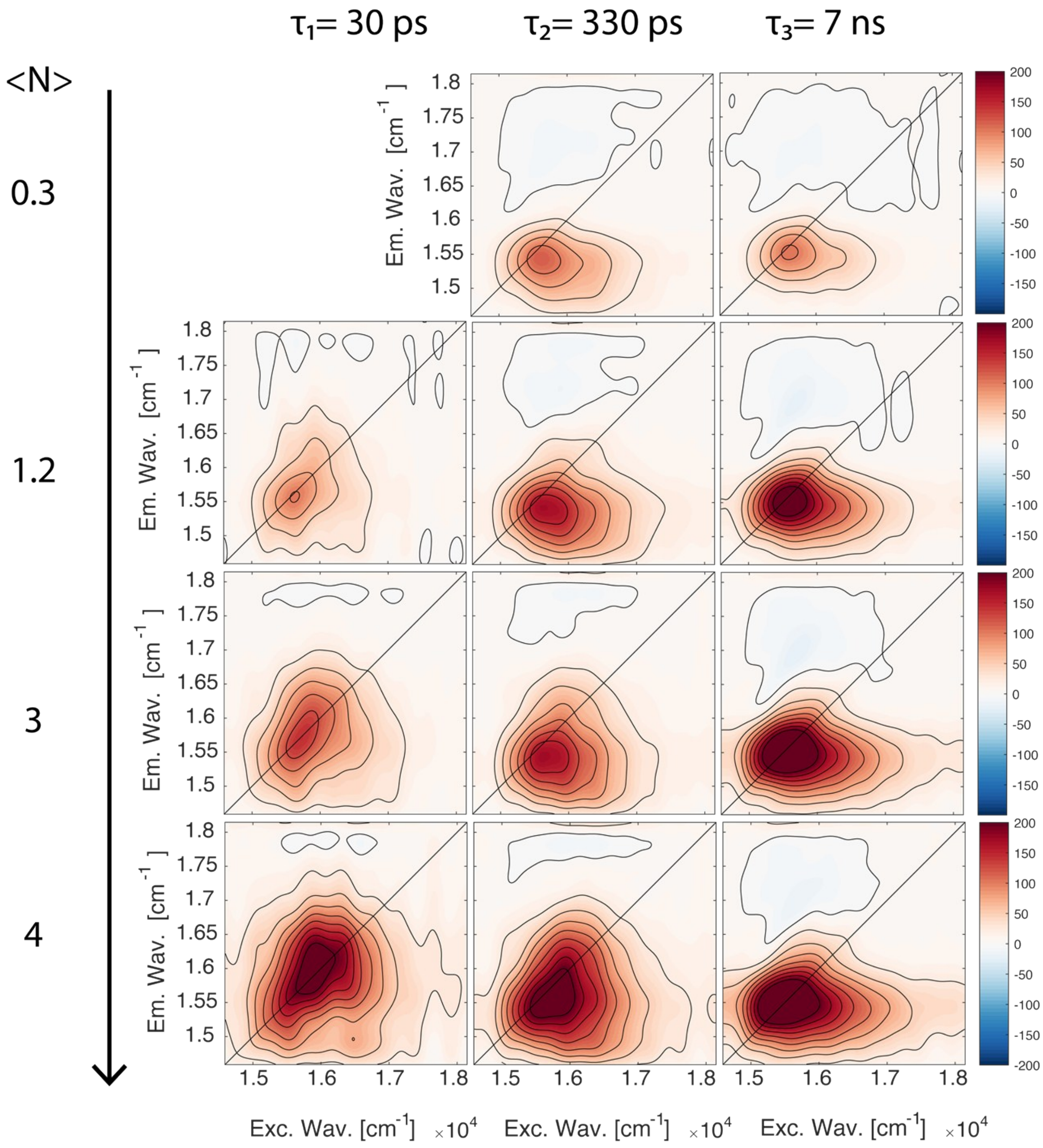

Figure SI_5- Global fitting results of 2DES-PP maps. Each row reports the DAS obtained at a fixed fluence, labeled with the average exciton number on the left. Hence each column reports the evolution of a specific DAS as a function of the fluence parameter.

Coherent Dynamics in 2DES-BC

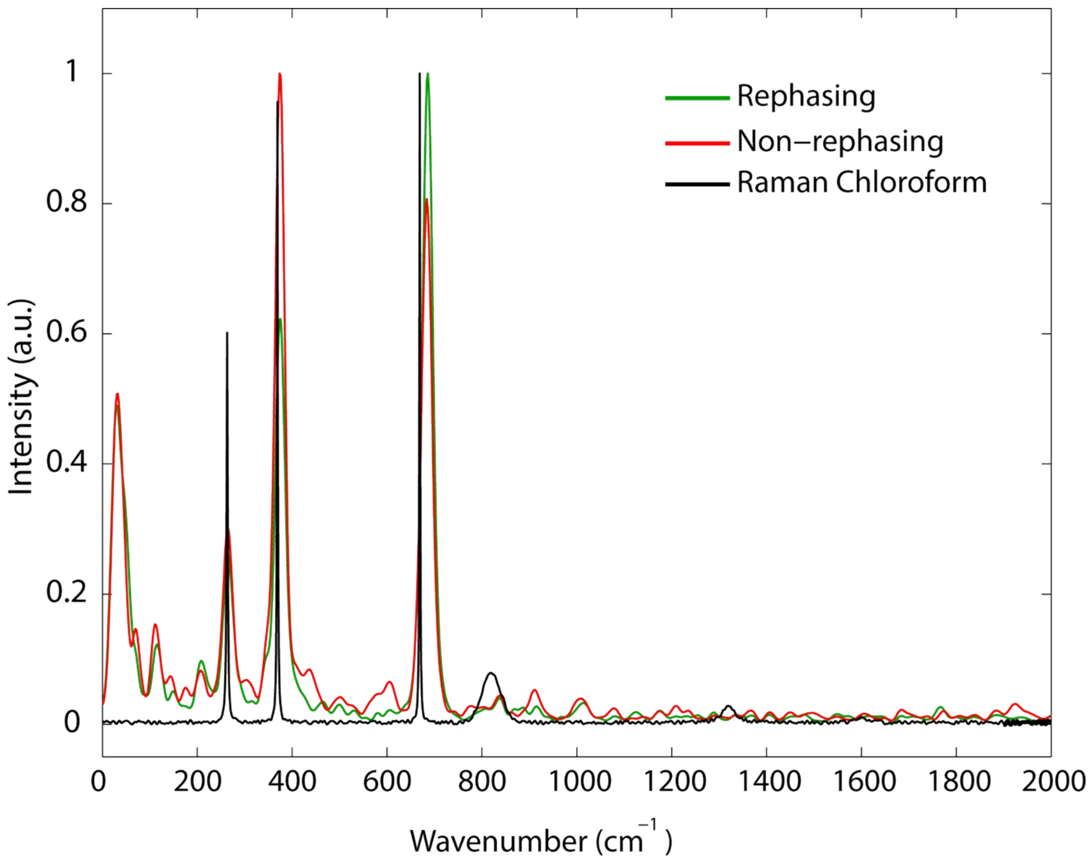

Figure SI_6- Fourier Transform spectra of the residual beating signals of the rephasing (green), non-rephasing (red) 2DES-BC maps, compared with the Raman spectrum of chloroform (black).

As described above, the global fitting methodology allows extracting both decaying and oscillating components. The analysis of 2DES-BC datasets revealed the presence of both coherent and incoherent dynamics along t_2. Hence, together with the non-oscillating exponential decay corresponding to the population relaxation described in the main text, we observed several oscillations suggesting the presence of coherent dynamics. However, since the third-order polarization of chloroform is particularly high, a substantial solvent contribution to oscillating signal via optical Kerr effect is expected. Applying a Fourier transform to the residuals of the fit, the power spectrum of the beating frequencies is obtained (Figure SI_6), that relates oscillation frequencies with their relative intensities. The comparison between the Raman spectrum of chloroform and beating spectra of rephasing and non-rephasing maps reveals that the major oscillating contributions (at $265,\ 375,\ 680\ cm^{-1}$) are associated with the non-resonant response of chloroform.

A closer look at the power spectra reveals the presence of weaker features not attributable to chloroform at around 30 and $210\ cm^{-1}$. These signals are in fair agreement with reported values for longitudinal acoustic (LA) and longitudinal optic (LO) phonon modes. However, the strong non-resonant response of the solvent conceals the role of these vibrations in the relaxation; future work will address this point.

Additional 2DES-PP measurements

We performed additional measurements with a redshifted excitation laser band, reported in Figure SI_7. The fitting of this second dataset provided results (time constants and amplitude distributions) in agreement with the first dataset reported in the main text, as shown in the DAS of Figure SI_8.

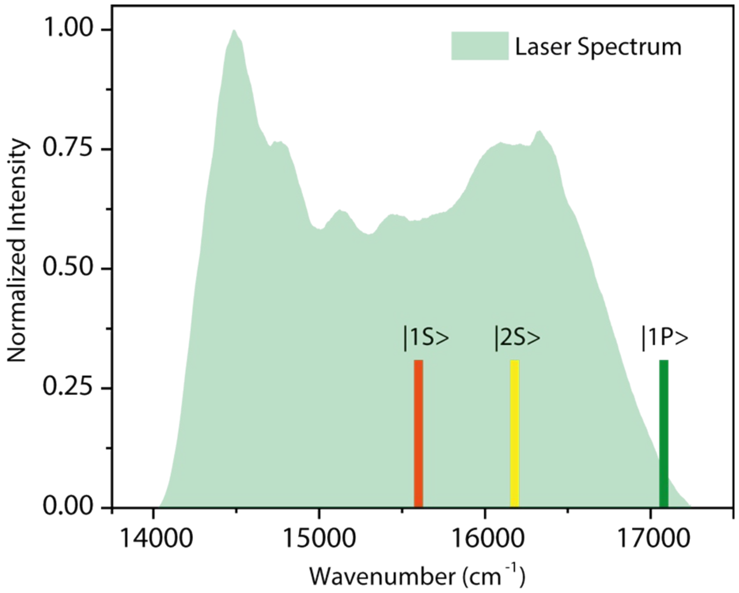

Figure SI_7- Laser spectrum used for additional 2DES-PP measurements and position of the main exciton peaks.

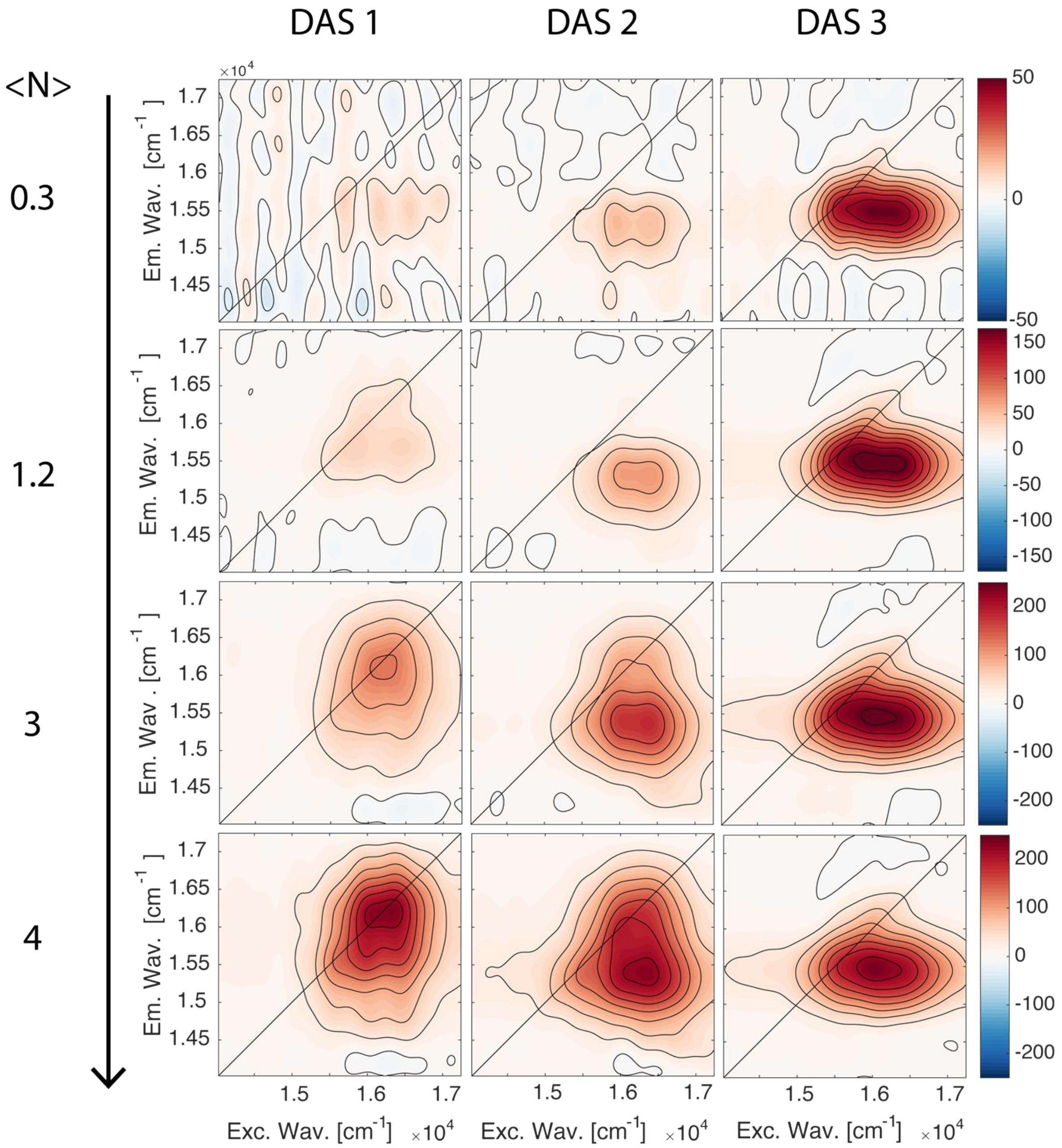

Figure SI_8- Global fitting results of 2DES-PP maps, obtained with a redshifted spectrum. Each row reports the DAS obtained at a fixed fluence, labeled with the average exciton number on the left. Hence each column reports the evolution of a specific DAS with the fluence parameter.

Bi-Exciton Binding Energy

To obtain a quantitative estimate of the bi-exciton binding energy in CdSe/ZnS QDs, we compared the spectral position of the peaks observed in the second and third DAS obtained by global analysis of the 2DES-PP dataset. As stated in the main text, we identify the second DAS (associated with the characteristic time $\tau = 330\, ps$) with the dynamics of BX stimulated emission signal. On the other hand, we assign the third DAS ($\tau = 7\, ns$) to the slower single exciton recombination. Accordingly, the two peaks are slightly redshifted along the emission frequency axis by a quantity, referred as bi-exciton binding energy Δ_{BX}.

To obtain a quantitative estimate of Δ_{BX}, we analyzed vertical slices of the DAS extracted at a fixed value of excitation energy corresponding to the $|1S\rangle$ energy ($15600\, cm^{-1}$). Figure SI_9 and Figure SI_10 report the comparison between the vertical slices at $15600\, cm^{-1}$ of the two DAS. Notably, the peak of DAS 2 is redshifted with respect to DAS 3 in both measurements. Hence, we could estimate an average value of $\Delta_{BX} = 190 \pm 10\, cm^{-1}$. This value is in agreement with previous studies based on TA spectroscopy and fluorescence spectroscopy, reporting values ranging from 120 to $350\, cm^{-1}$. [8,9]

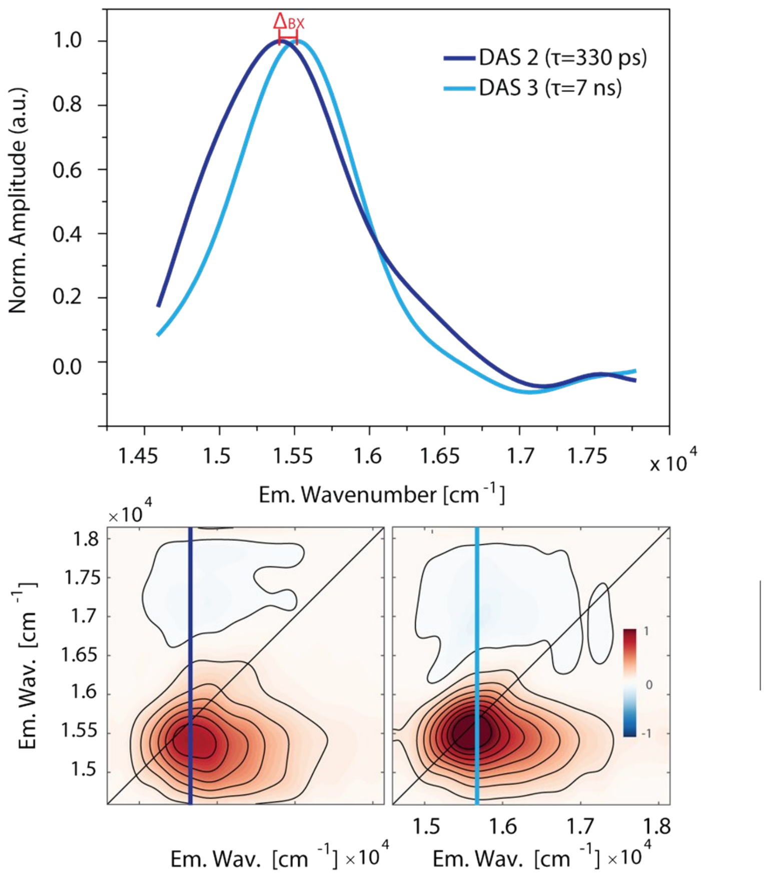

Figure SI_9- Comparison between vertical slices of DAS 2 and DAS 3 extracted at a fixed value of excitation energy. DAS 2 and DAS 3 are the results of the global fit of the first set of 2DES-PP measures, as shown in (Figure SI_5).

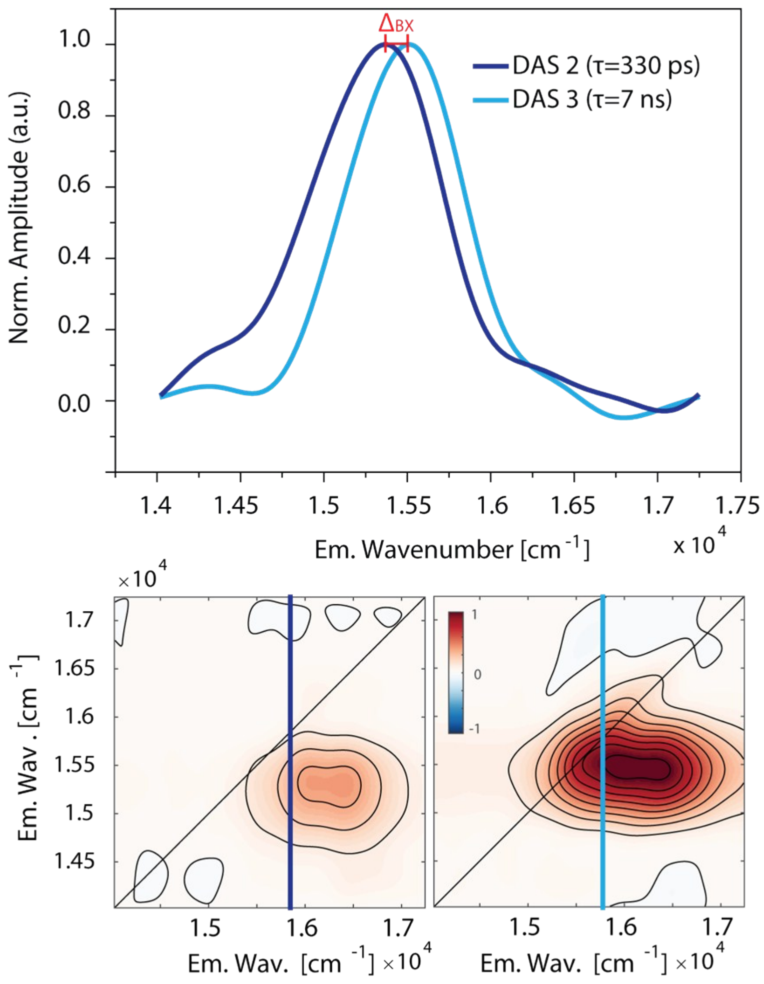

Figure SI_10- Same as Figure SI_9, but referred to DAS 2 and DAS 3 obtained from the global fit of the second set of 2DES_PP measures, as shown in Figure SI_8.

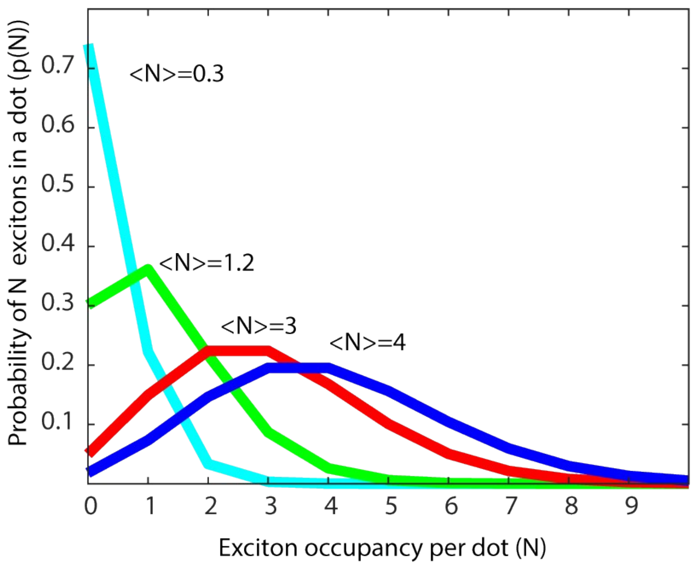

Figure SI_11- Poisson distributions with the average values $\langle N \rangle$ associated with the 2DES-PP measurements.

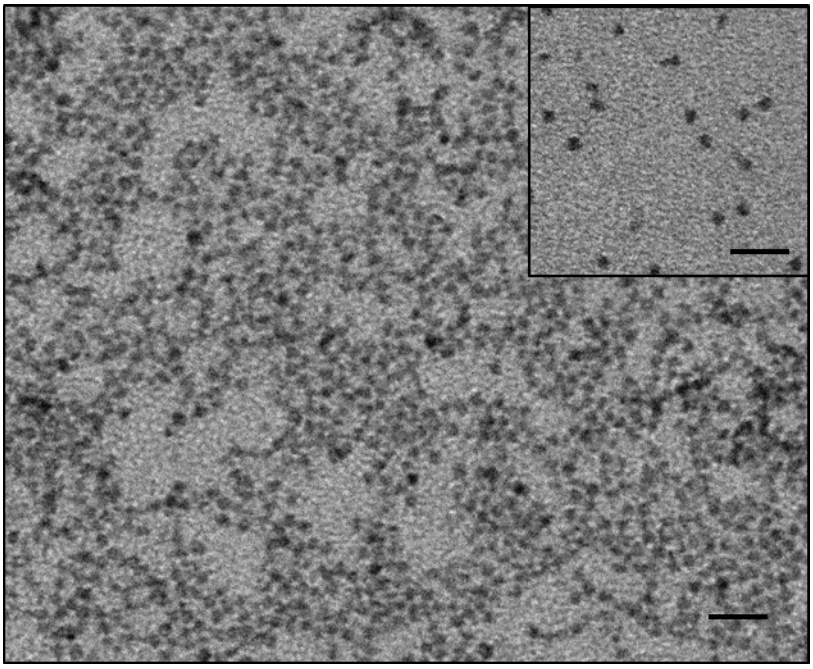

Figure SI_12 Transmission electron microscopy (TEM) micrographs of CdSe/ZnS QDs obtained by a JEOL JEM-1011 microscope operating at 100 kV. The specimens were prepared by depositing few drops of chloroform solutions of QDs onto a carbon-coated copper grid and allowing the solvent to evaporate (scale bars 20 nm).

REFERENCES

1. E. Fanizza, M. Altomare, A. E. Di Mauro, T. Del Sole, M. Corricelli, N. Depalo, R. Comparelli, A. Agostiano, M. Striccoli, M. L. Curri, *Langmuir* 2012, **28**, 5964-5974
2. E. Fanizza, C. Urso, V. Pinto, A. Cardone, R. Ragni, N. Depalo, M. L. Curri, A. Agostiano, G. M. Farinola, M. Striccoli, *J .Mater. Chem C,* 2014, **2**, 5286-5291.
3. L. Bolzonello, A. Volpato, E. Meneghin, E. Collini, *J. Opt. Soc. Am. B* 2017, **34**, 1223-1233.
4. Fleming, G. R. Chemical applications of ultrafast spectroscopy. *Oxford University Press*, Incorporated: **1986**.
5. S. Yan, H.- S. Tan, *Chem. Phys.* 2009, **360**, 110-115
6. Z. Zhang, K. L. Wells, E. W. J. Hyland, H.-S. Tan, *Chem. Phys. Lett,*. 2012, **550**, 156–161.
7. A. Volpato, L. Bolzonello, E. Meneghin, E. Collini, *Opt. Express* 2016, ***24***, 24773-24785.
8. V. I. Klimov, *Annu. Rev. Phys. Chem*. 2007, **58***,* 635-673.
9. S. L. Sewall, R. R. Cooney, K. E. H. Anderson, E. A. Dias, D. M. Sagar, P. Kambhampati, *J. Chem. Phys*., 2008, **129**, 084701.
